# A New *Epi*-neoverrucosane-type Diterpenoid from the Liverwort *Pleurozia subinflata* in Borneo

**DOI:** 10.1007/s13659-020-00232-6

**Published:** 2020-02-15

**Authors:** Takashi Kamada, Mary Lyn Johanis, Shean-Yeaw Ng, Chin-Soon Phan, Monica Suleiman, Charles S. Vairappan

**Affiliations:** 1 Department of Materials and Life Science, Faculty of Science and Technology, Shizuoka Institute of Science and Technology, 2200-2 Toyosawa, Fukuroi, Shizuoka 437-8555 Japan; 2 Institute for Tropical Biology and Conservation, Universiti Malaysia Sabah, 88400 Kota Kinabalu, Sabah Malaysia

**Keywords:** *Epi*-neoverrucosane, Diterpenoid, *Pleurozia subinflata*, Liverwort, Borneo

## Abstract

**Abstract:**

New bioactive 13-*epi*-neoverrucosane diterpenoid, 5β-acetoxy-13-*epi*-neoverrucosanic acid (**1**) along with three known secondary metabolites, 13-*epi*-neoverrucosan-5β-ol (**2**), chelodane (**3**) and (*E*)-β-farnesene (**4**) were isolated from the MeOH extract of east Malaysia’s liverwort *Pleurozia subinflata*. The chemical structure of new compound was elucidated by the analyses of its spectroscopic data (FTIR, NMR and HR-ESI-MS). These *epi*-neoverrucosane-type compounds seem to be notable chemosystematic markers for *P. subinflata* in Borneo. Compound **3** was widespread in marine sponges however this is the first record for **3** to be found in liverwort. These metabolites were tested for their antifungal potentials against selected fungi from the marine environment. Compound **1** exhibited effective antifungal activity against *Lagenidium thermophilum*.

**Graphic Abstract:**

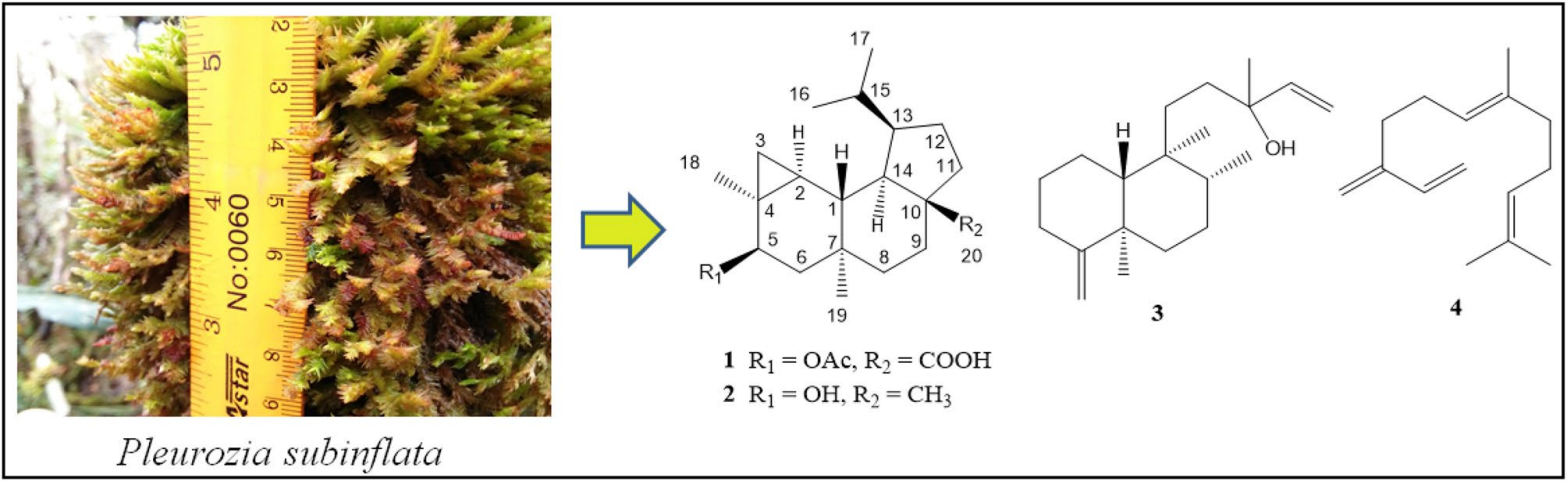

**Electronic supplementary material:**

The online version of this article (10.1007/s13659-020-00232-6) contains supplementary material, which is available to authorized users.

## Introduction

**Fig. 1:**
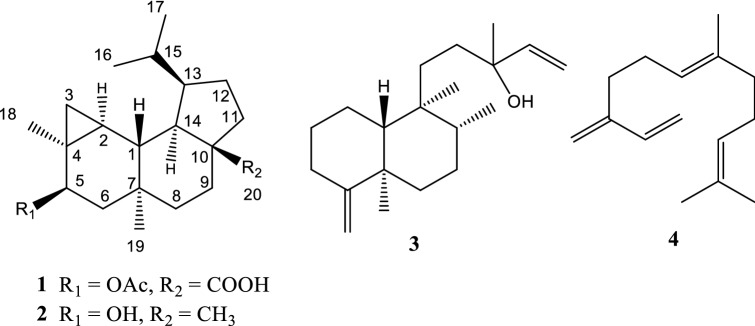
Chemical structures of **1**–**4**

Liverworts are the largest group of pioneering land plants which arose during the adaptation of plants from marine to terrestrial environment [1]. They produced terpenoids and/or aromatic compounds as their major lipophilic constituents [2–5]. Many types of sesquiterpenoids from liverworts are the enantiomer to those metabolites from higher plants [6]. While, diterpenoids such as clerodanes, dolabellanes, fusicoccanes, kauranes, labdanes, pimaranes and others were found in numerous liverworts [6]. Recently several bioactive cyathane diterpenoids were discovered [7, 8]. Cyathane is precursor structure for biosynthesis of the verrucosane-type diterpenoid, a fused 3,6,6,5-tetracyclic carbon skeleton [9]. First verrucosane diterpenoid was isolated from *Mylia verrucosa* [10]. Later, some analogs such as neoverrucosane-, homoverrucosane-, *epi*-neoverrucosane- and *epi*-homoverrucosane-type were reported [11–13]. The latest *epi*-neoverrucosane analog was reported in 2013 [14]. Hereby, we report yet another new *epi*-neoverrucosane diterpenoid, 5β-acetoxy-13-*epi*-neoverrucosanic acid (**1**) was isolated, along with three known secondary metabolites, 13-*epi*-neoverrucosan-5β-ol (**2**), chelodane (**3**) and (*E*)-β-farnesene (**4**) from east Malaysia’s liverwort *Pleurozia subinflata* (Fig. 1).

Besides, liverworts have long been used as traditional medicine by the indigenous people in some parts of China. In the past half-century, Prof. Yoshinori Asakawa (Tokushima Bunri University, Japan) has begun to study the chemical composition of liverworts collected from Asia, Europe and South America, many of which have reported to have diverse chemical structures and exhibited numerous biological activities [2, 6]. Thus, research focusing on the biological activity of liverwort and industrial use are significant. Our research examined the antifungal effects of the four isolated compounds against selected fungi separated marine organisms.

## Results, Discussion and Conclusion

Compound **1** was isolated as colorless oil and analyzed for the molecular formula C_22_H_34_O_4_ by HR-ESI-MS [M − H]^−^ ion at *m/z* 361.2391. The ^13^C and ^1^H NMR data (Table 1) indicated the presence of an isopropyl unit at *δ*_C_ 31.1, 23.2 and 22.3; *δ*_H_ 1.51–1.55, 1.00 and 0.78, one carboxylic carbon at *δ*_C_ 183.0, an acetoxy unit at *δ*_C_ 171.5, 21.5; *δ*_H_ 2.04, one oxygenated methine at *δ*_C_ 75.1; *δ*_H_ 5.28, two tertiary methyls at *δ*_C_ 25.5 and 16.4; *δ*_H_ 1.12 and 0.87, six methylenes, four methines, and three quaternary carbons which corresponding well to HSQC spectrum. Based on these findings, six degrees of unsaturation was calculated from HR-ESI-MS, and it attributed to two carbonyl units and one tetracyclic ring for **1**.

**Table 1:** ^1^H and ^13^C NMR (600 and 150 MHz) data of **1** (in CDCl_3_, *δ* in ppm, *J* in Hz).

No	*δ*_C_	*δ*_H_
1	44.1	2.20 (1H, dd, *J* = 4.3, 13.1)
2	26.7	0.65 (1H, ddd, *J* = 4.1, 4.1, 8.3)
3	21.8	0.69 (1H, dd, *J* = 4.3, 8.3)
		0.56 (1H, dd, *J* = 4.3, 4.3)
4	20.1	
5	75.1	5.28 (1H, dd, *J* = 7.6, 10.7)
6	42.3	1.76 (1H, dd, *J* = 7.6, 12.5)
		0.84 (1H, m)
7	36.8	
8	34.9	1.51–1.55 (1H, m)
		1.09–1.11 (1H, m)
9	32.3	1.81–1.84 (1H, m)
		1.59–1.62 (1H, m)
10	52.0	
11	36.3	2.45 (1H, ddd, *J* = 1.4, 8.9, 11.6)
		1.21–1.23 (1H, m)
12	27.5	1.81–1.84 (1H, m)
		1.51–1.55 (1H, m)
13	45.3	1.88 (1H, m)
14	51.7	1.67 (1H, dd, *J* = 7.6, 13.1)
15	31.1	1.51–1.55 (1H, m)
16	22.3	0.78 (3H, d, *J* = 6.9)
17	23.2	1.00 (3H, d, *J* = 6.9)
18	25.5	1.12 (3H, s)
19	16.4	0.87 (3H, s)
20	183.0	
5-OAc	171.5	
	21.5	2.04 (3H, s)

**Fig. 2:**
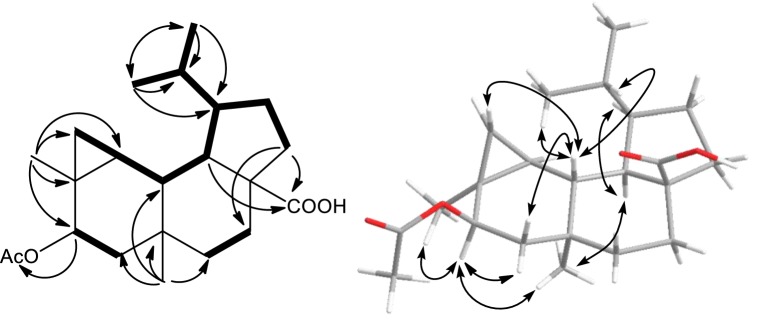
^1^H–^1^H COSY, key HMBC and NOE correlations of **1**

The ^1^H–^1^H COSY experiment revealed the spin systems as depicted by the bold lines in Fig. 2. In the HMBC spectrum, the three-bond correlations of H_3_–C(16) and H_3_–C(17) to the opposite carbons C(17) and C(16), and to C(13) and C(15), allowed the placement of isopropyl unit at C(13) which was further confirmed by ^1^H–^1^H COSY. The acetoxy unit at C(5) was confirmed by HMBC correlations between H–C(5) to 5-OAc. The downfield shifted of ^13^C and ^1^H NMR at C(5) further supported this deduction. The HMBC correlations of H_2_–C(11) to C(20); and H–C(14) to C(20) suggested the carboxylic carbon at C(10). These findings together with HMBC correlations of H_3_–C(18) to C(2), C(3), C(4) and C(5); and H_3_–C(19) to C(1), C(6), C(7) and C(8) permitted establishment for the planar structure of **1** (Fig. 2).

**Fig. 3:**
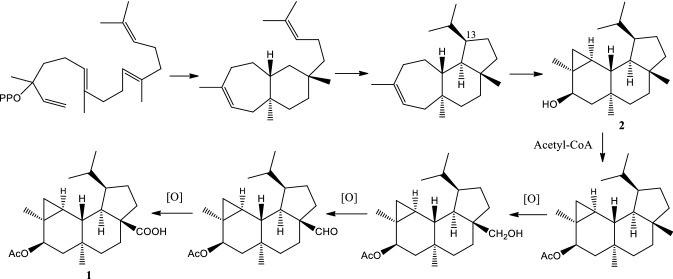
Proposed biosynthetic pathway of **1**

The relative stereochemistry of **1** was deduced from the NOESY correlations (Fig. 2) and comparison of its chemical shift, coupling constants and NOE correlations with those of known analogs [10–14]. The NOE correlations of H–C(5) to H_2_–C(6α) (*δ*_H_ 1.76), H_3_–C(18) and H_3_–C(19); and H–C(14) to H_3_–C(19) have suggested H–C(5), H–C(14), H_3_–C(18) and H_3_–C(19) on α relative configuration. While, NOE correlations of H–C(1) to H_2_–C(3β) (*δ*_H_ 0.56) and H_2_–C(6β) (*δ*_H_ 0.84) showed H–C(1) must be on another face, β relative configuration. The earlier NOE cross peak of H–C(1) to H_2_–C(3) (*δ*_H_ 0.56) has led to the assignment of H_2_–C(3) (*δ*_H_ 0.56) on β configuration, therefore H_2_–C(3) (*δ*_H_ 0.69) must be on α configuration. With this finding, the configuration at H–C(2) can be assigned based on vicinal coupling constants of cyclopropane unit between H–C(2) and H_2_–C(3α) (^3^*J*_2-3α_ = 8.3 Hz) and between H–C(2) and H_2_–C(3β) (^3^*J*_2-3β_ = 4.3 Hz). These coupling constant values suggested H–C(2) has a *cis* relationship with H_2_–C(3α) within the cyclopropane unit, therefore α configuration was assigned for H–C(2). While, the carboxyl unit at C(10) was assigned on β configuration due to a *trans*-fused at C/D ring junction. Thus, the relative configurations of 1R*, 2S*, 4S*, 5R*, 7S*, 10S* and 14R* were determined as identical to those of known analogs of neoverrucosane and *epi*-neoverrucosane [10–14]. To distinguish *epi*-neoverrucosane from neoverrucosane, the NOE correlations of H–C(1) to H–C(15) and H_3_–C(17); and H–C(13) to H–C(14), showed 13-isopropyl unit to H–C(14) has a *trans* configuration, suggested a *epi*-neoverrucosane. Furthermore, similar NOE correlations of H–C(1) to H–C(15); and H–C(13) to H–C(14) were observed in 12-acetoxy-13-*epi*-neoverrucosann-5-one [14]. On the contrary, these NOE were not observed in neoverrucosane-type, neoverrucosan-5β,9β-diol, instead H–C(13) to H–C(20) was detected [15]. Thus, the structure **1** was established without confusion. The configuration of isopropyl unit at C(13) generated during formation of tricyclic system (Fig. 3) determined the biosynthesis of neoverrucosane or *epi*-neoverrucosane [9]. To the best of our knowledge, compound **1** was considered as first discovery of 13-*epi*-neoverrucosane that containing a carboxyl moiety or even among related skeletons such as verrucosane and neoverrucosane. The methyl at C(20) of **1** might have followed a three-step oxidation, via a hydroxyl and carbonyl, to the corresponding carboxylic acid [16], as shown in the purposed biosynthetic pathway (Fig. 3).

The known compounds were identified as 13-*epi*-neoverrucosan-5β-ol (**2**) [11], chelodane (**3**) [17], and (*E*)- β-farnesene (**4**) [18], after compared its spectroscopic data with published literatures. The tetracyclic diterpenes are relatively rare in nature, and mainly found in the species of *Plagiochila*, *Jamesoniella* and *Fossombronia* [6]. However, we found *epi*-neoverrucosane-type diterpene derivatives (**1** and **2**) from east Malaysia’s liverwort, *Pleurozia subinflata*. These secondary metabolites seem to be the good chemosystematic markers for *P. subinflata* in Borneo. Compound **3** was widespread in marine sponges such as *Chelonaplysilla erecta*, *Raspailia* sp. and even in *Sigmosceptrella* sp. [17, 19, 20]. However, this is the first record for **3** found in liverwort. Compound **4** was the most common farnesane-type sesquiterpene in liverworts. It was distributed throughout more than 20 Jungermanniales and Pleuroziales species including *Pleurozia* [6].

Compounds **1**–**4** were evaluated its biological potentials against fungal strains isolated from the Bornean ocean, *Lagenidium thermophilum* IPMB 1401, *Haliphthoros sabahensis* IPMB 1402, *Fusarium moniliforme* NJM 8995, *Fusarium oxysporum* NJM 0179, *Fusarium solani* NJM 8996 and *Ochroconis humicola* NJM 1503 (Table 2). The minimum inhibition concentration (MIC) values of compound **1** against *L. thermophilum* and *H. sabahensis* were 12.5 and 50 μg/mL, respectively. While, compounds **2** and **3** showed MIC values of 100 μg/mL against *L. thermophilum*. Compound **4** was inactive (> 100 μg/mL) against the tested fungi.

**Table 2:** The MIC (μg/mL) of **1**–**4** against six fungal strains

Strains	MIC (µg/mL)			
	**1**	**2**	**3**	**4**
*L. thermophilum*	12.5	100.0	100.0	> 100.0
*H. sabahensis*	50.0	> 100.0	> 100.0	> 100.0
*F. moniliforme*	> 100.0	> 100.0	> 100.0	> 100.0
*F. oxysporum*	> 100.0	> 100.0	> 100.0	> 100.0
*F. solani*	> 100.0	> 100.0	> 100.0	> 100.0
*O. humicola*	> 100.0	> 100.0	> 100.0	> 100.0

Positive control: itraconazole MIC 2.0 µg mL^−1^

In conclusion, this is the first time ever since 2013 of the last *epi*-neoverrucosane being discovered from nature. A new 13-*epi*-neoverrucosane diterpenoid, 5β-acetoxy-13-*epi*-neoverrucosanic acid (**1**) along with three known secondary metabolites, 13-*epi*-neoverrucosan-5β-ol (**2**), chelodane (**3**) and (*E*)-β-farnesene (**4**) were found in east Malaysia’s liverwort *Pleurozia subinflata*. Compound **1** exhibited effective antifungal activity (MIC values of 12.5 μg/mL) against *Lagenidium thermophilum*.

## Experimental Section

### General Experimental Procedures

Optical rotation was taken on the automatic polarimeter (AUTOPOL IV automatic polarimeter) in chloroform solutions at 28 °C. IR spectrum was recorded on the FTIR spectroscopy (Perkin Elmer). NMR spectra were recorded on the 600 MHz FT-NMR (Jeol) using deuterated chloroform (CDCl_3_) with tetramethylsilane (TMS) as the internal standard. MS spectra were obtained using LC-ESI-IT-TOF-MS (Shimadzu). For preparative TLC, Merck Kieselgel 60 F_254_ was used and Kieselgel 60 was used for column chromatography. Purification was performed using high performance liquid chromatography (LC-10 AT, Shimadzu) equipped with UV detector.

### Biological Materials

Specimens of *P. subinflata (M. Suleiman & S.-Y. Ng 5946)* were collected from Mount Trus Madi (5° 33′ 13.1″ N, 116° 30′ 41.9″ E), Sabah, Malaysia in August 2015. The specimens were identified based on external morphology by the fifth author. A voucher specimen (BORHB0026) is deposited in the BORNEENSIS Herbarium at Institute for Tropical Biology and Conservation (ITBC), Univeristi Malaysia Sabah (UMS).

### Extraction and Isolation

The air-dried liverwort specimens (42 g) were extracted using 100% methanol (MeOH) (1.0 L × 3 each for two days). The crude extract was partitioned between distilled water (150 mL) and ethyl acetate (EtOAc) (50 mL × 3). After removal of the organic solvent, the EtOAc fraction (500 mg) was chromatographed on a Si gel column using hexane (Hex) and EtOAc system as eluent with increasing polarity (Hex/EtOAc: 9:1, 8:2, 7:3, 5:5, 100% EtOAc) to yield five fractions, 1–5. Fraction 2 (76 mg) was subjected to repeated preparative TLC with toluene to yield **2** (8.8 mg), **3** (7.4 mg) and **4** (15.4 mg). Fraction 3 (60 mg) was subjected to repeated preparative TLC with hexane/EtOAc: 7:3, and the resulted sub-fraction was further purified by semi-preparative high performance liquid chromatography (HPLC) to yield **1** (12.8 mg). The isolation was operated using a reverse phase C_18_ column (5 μm, 10 mm × 250 mm) measured at UV wavelength of 210 nm under gradient elution with the following conditions: 40–100% acetonitrile (MeCN)/H_2_O.

#### 5β-Acetoxy-13-*Epi*-neoverrucosanic Acid (1)

Colorless oil; [α]_D_^28.0^ + 67.8 (*c* = 0.5, CHCl_3_); IR ν_max_ 3488, 3060, 1735 and 1712 cm^−1^; ^1^H NMR (CDCl_3_, 600 MHz) *δ* 5.28 (1H, dd, *J* = 7.6, 10.7 Hz, H-5), 2.45 (1H, ddd, *J* = 1.4, 8.9, 11.6 Hz, H-11α), 2.20 (1H, dd, *J* = 4.3, 13.1 Hz, H-1), 2.04 (3H, s, OAc), 1.88 (1H, m, H-13), 1.81–1.84 (1H, m, H-12α), 1.81–1.84 (1H, m, H-9α), 1.76 (1H, dd, *J* = 7.6, 12.5 Hz, H-6α), 1.67 (1H, dd, *J* = 7.6, 13.1 Hz, H-14), 1.59–1.62 (1H, m, H-9β), 1.51–1.55 (1H, m, H-8α), 1.51–1.55 (1H, m, H-12β), 1.51–1.55 (1H, m, H-15), 1.21–1.23 (1H, m, H-11β), 1.12 (3H, s, H-18), 1.09–1.11 (1H, m, H-8β), 1.00 (3H, d, *J* = 6.9 Hz, H-17), 0.87 (3H, s, H-19), 0.84 (1H, m, H-6β), 0.78 (3H, d, *J* = 6.9 Hz, H-16), 0.69 (1H, dd, *J* = 4.3, 8.3 Hz, H-3α), 0.65 (1H, ddd, *J* = 4.1, 4.1, 8.3, H-2), 0.56 (1H, dd, *J* = 4.3, 4.3 Hz, H-3β); ^13^C NMR (CDCl_3_, 150 MHz) *δ* 183.0 (C, C-20), 171.5 (C, OAc), 75.1 (CH, C-5), 52.0 (C, C-10), 51.7 (CH, C-14), 45.3 (CH, C-13), 44.1 (CH, C-1), 42.3 (CH_2_, C-6), 36.8 (C, C-7), 36.3 (CH_2_, C-11), 34.9 (CH_2_, C-8), 32.3 (CH_2_, C-9), 31.1 (CH, C-15), 27.5 (CH_2_, C-12), 26.7 (CH, C-2), 25.5 (CH_3_, C-18), 23.2 (CH_3_, C-17), 22.3 (CH_3_, C-16), 21.8 (CH_2_, C-3), 21.5 (CH_3_, OAc), 20.1 (C, C-4), 16.4 (CH_3_, C-19); negative ion HRESIMS: *m/z* 361.2391 (calcd for C_22_H_33_O_4_ [M–H]^−^, 361.2384).

### Antifungal Assay

The minimum inhibitory concentration (MIC) of the fungistatic on hyphae was performed by incorporating the pure compound solutions (12.5, 25.0, 50.0, 100.0 μg/mL) onto PYGS agar in a petri dish followed by inoculation of six tested fungal strains [21–23]. The MIC was determined visually as the lowest concentration showing no hyphal growth after they were incubated at 25 °C for 7 days.

## Electronic supplementary material

Below is the link to the electronic supplementary material.

Supplementary file1 (DOCX 594 kb)
