# Supplementary material for: A New Epi-neoverrucosane-type Diterpenoid from the Liverwort Pleurozia subinflata in Borneo
Source: Nat Prod Bioprospect. 2020 Feb 15;10(1):51–6. doi: 10.1007/s13659-020-00232-6 (PMC7046843; doi:10.1007/s13659-020-00232-6)

Supplementary Materials

| **A new *epi*-neoverrucosane-type diterpenoid from**  **the liverwort *Pleurozia subinflata* in Borneo**  Takashi Kamada,*^,a,b^ Mary Lyn Johanis,^b^ Shean-Yeaw Ng,^b^ Chin-Soon Phan,^b^  Monica Suleiman,^b^ and Charles S. Vairappan^b^  ^a^*Department of Materials and Life Science, Faculty of Science and Technology, Shizuoka Institute of Science and Technology, 2200-2 Toyosawa, Fukuroi, Shizuoka 437-8555, Japan.*  ^b^Institute for Tropical Biology and Conservation, Universiti Malaysia Sabah, 88400 Kota Kinabalu, Sabah, Malaysia.  E-mail: takashi.kamada800@gmail.com  **Supplementary Materials**  **Figure S1.** ^1^H NMR spectrum of compound **1** in CDCl_3_ (600 MHz).  **Figure S2.** ^13^C NMR spectrum of compound **1** in CDCl_3_ (150 MHz).  **Figure S3.** HSQC spectrum of compound **1** in CDCl_3_.  **Figure S4.** ^1^H-^1^H COSY spectrum of compound **1** in CDCl_3_.  **Figure S5.** HMBC spectrum of compound **1** in CDCl_3_.  **Figure S6.** NOESY spectrum of compound **1** in CDCl_3_.  **Figure S7.** HR-ESI-MS spectrum of compound **1**. |
| --- |

**Figure S1.** ^1^H NMR spectrum of compound **1** in CDCl_3_ (600 MHz).


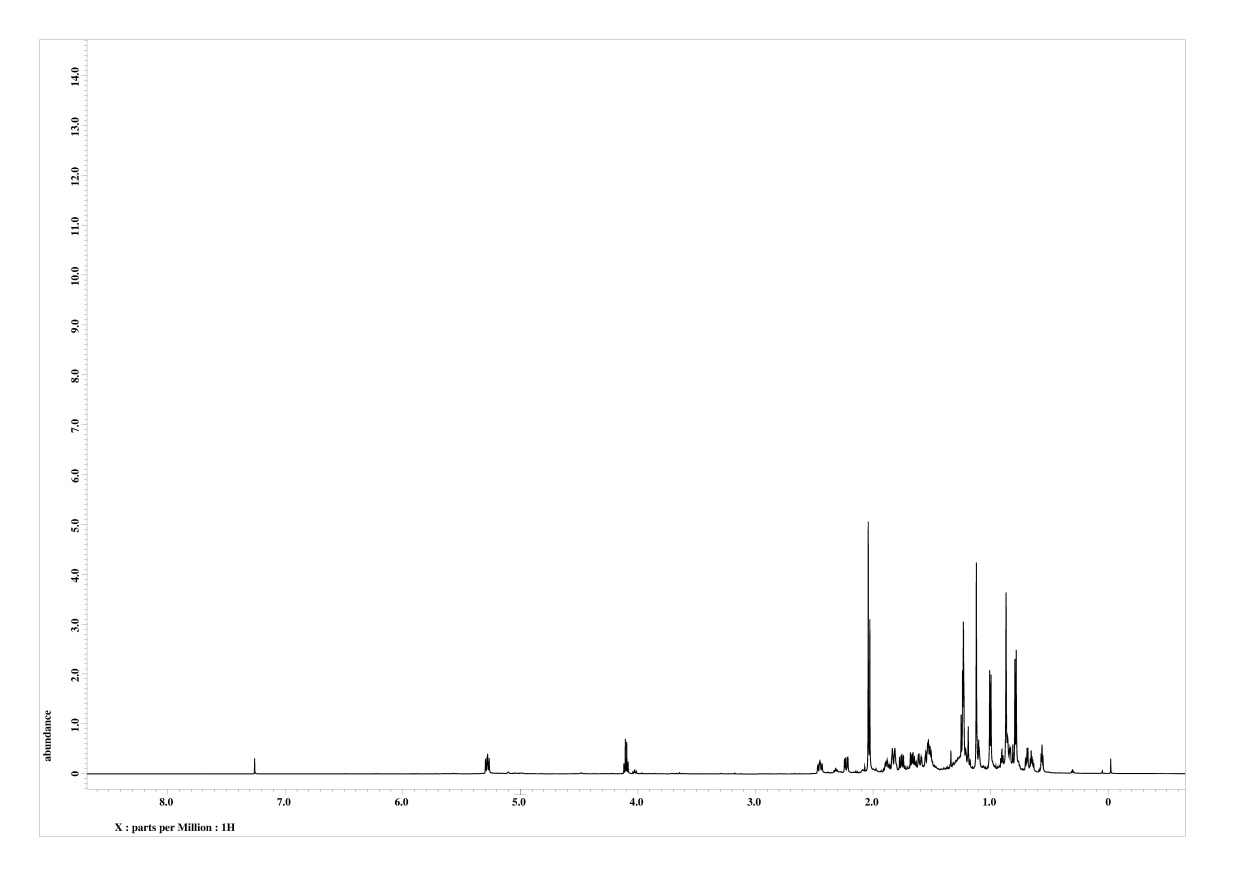


**Figure S2.** ^13^C NMR spectrum of compound **1** in CDCl_3_ (150 MHz).


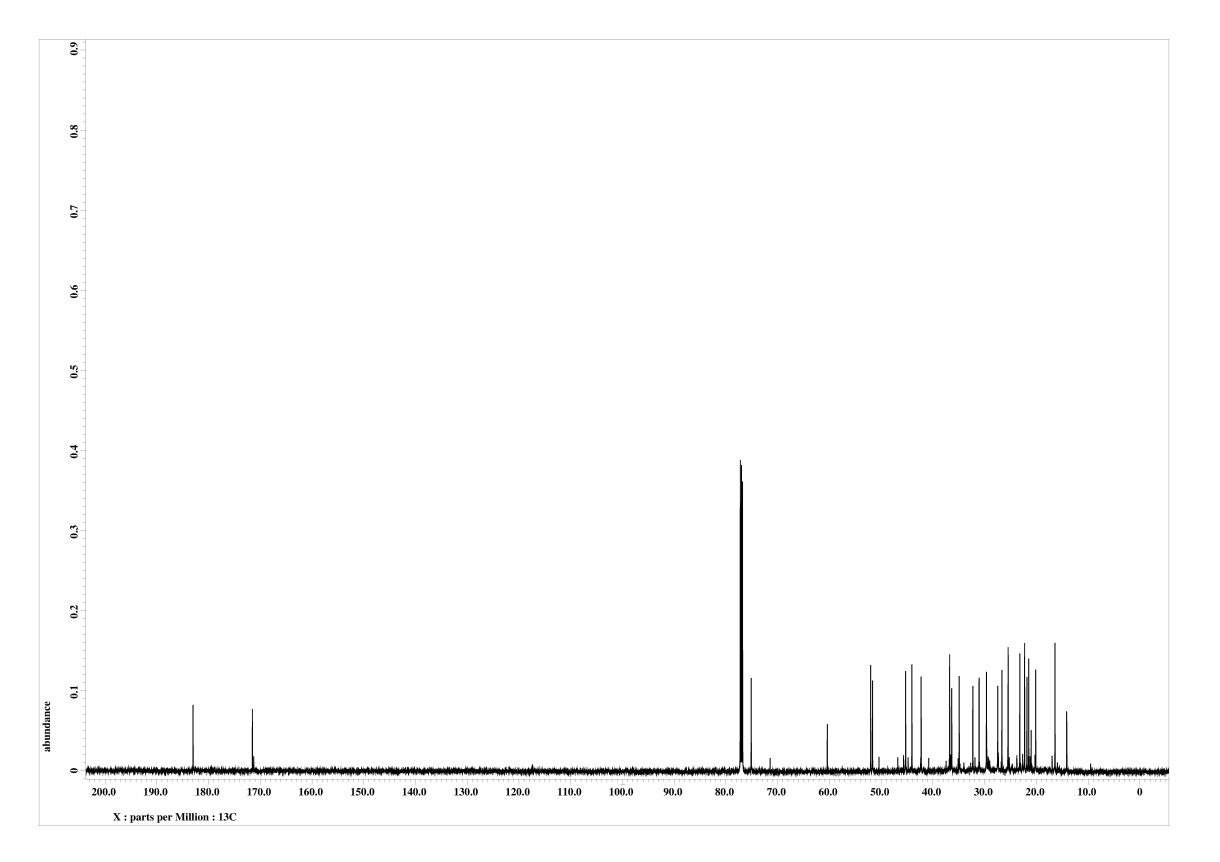


**Figure S3.** HSQC spectrum of compound **1** in CDCl_3_.


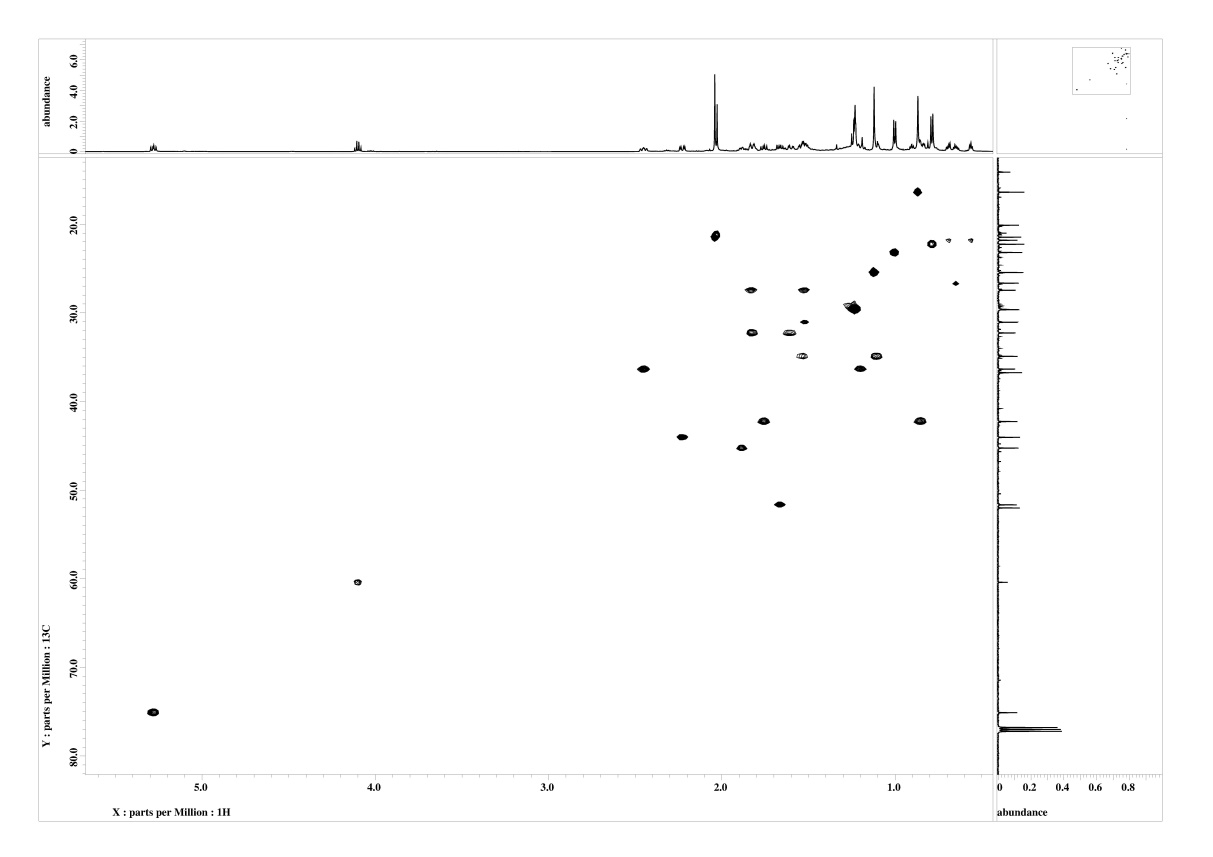


**Figure S4.** ^1^H-^1^H COSY spectrum of compound **1** in CDCl_3_.


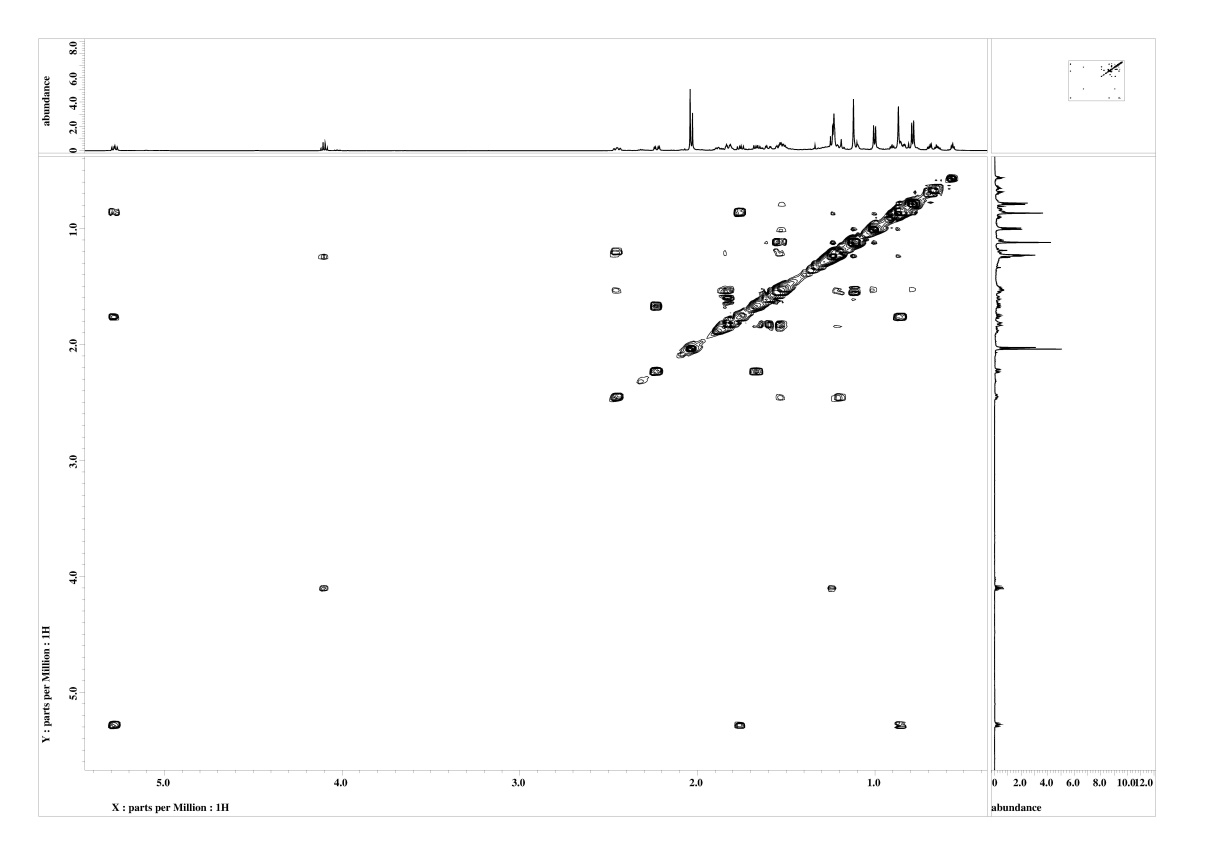


**Figure S5.** HMBC spectrum of compound **1** in CDCl_3_.


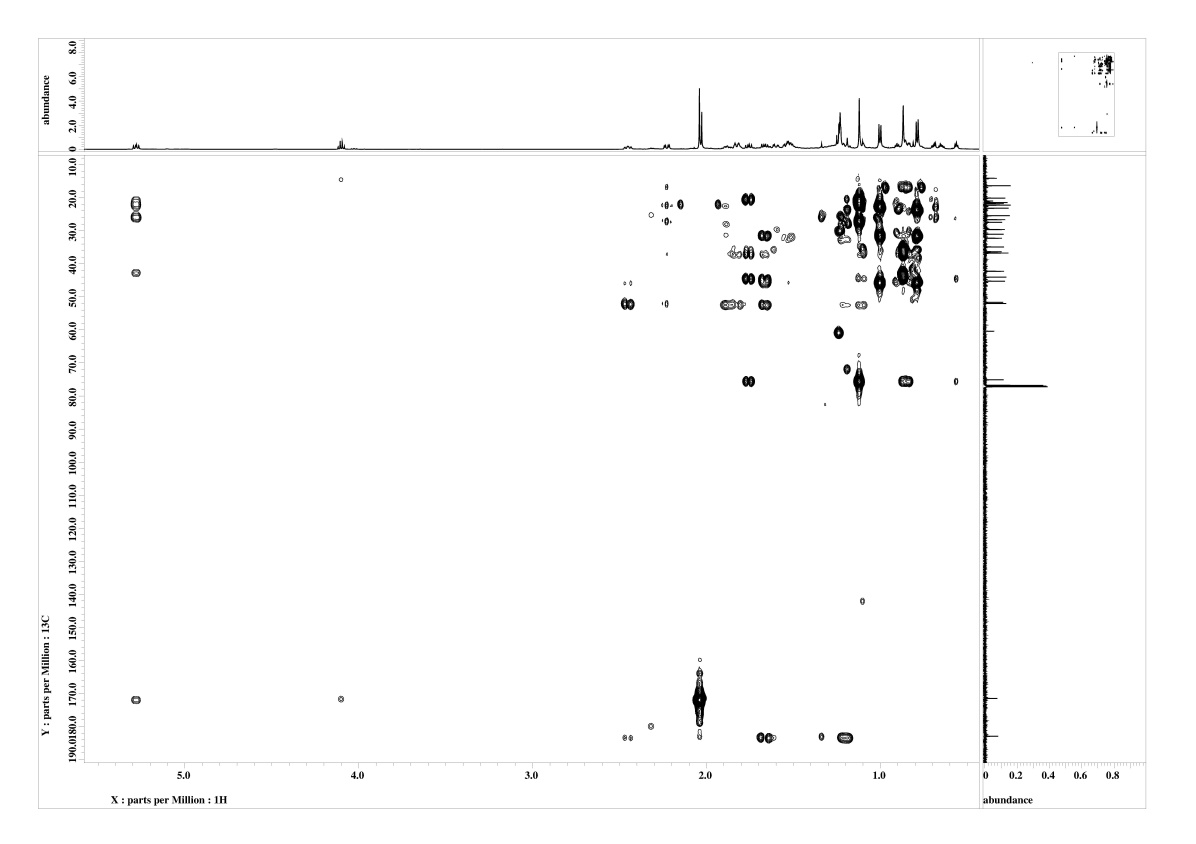


**Figure S6.** NOESY spectrum of compound **1** in CDCl_3_.


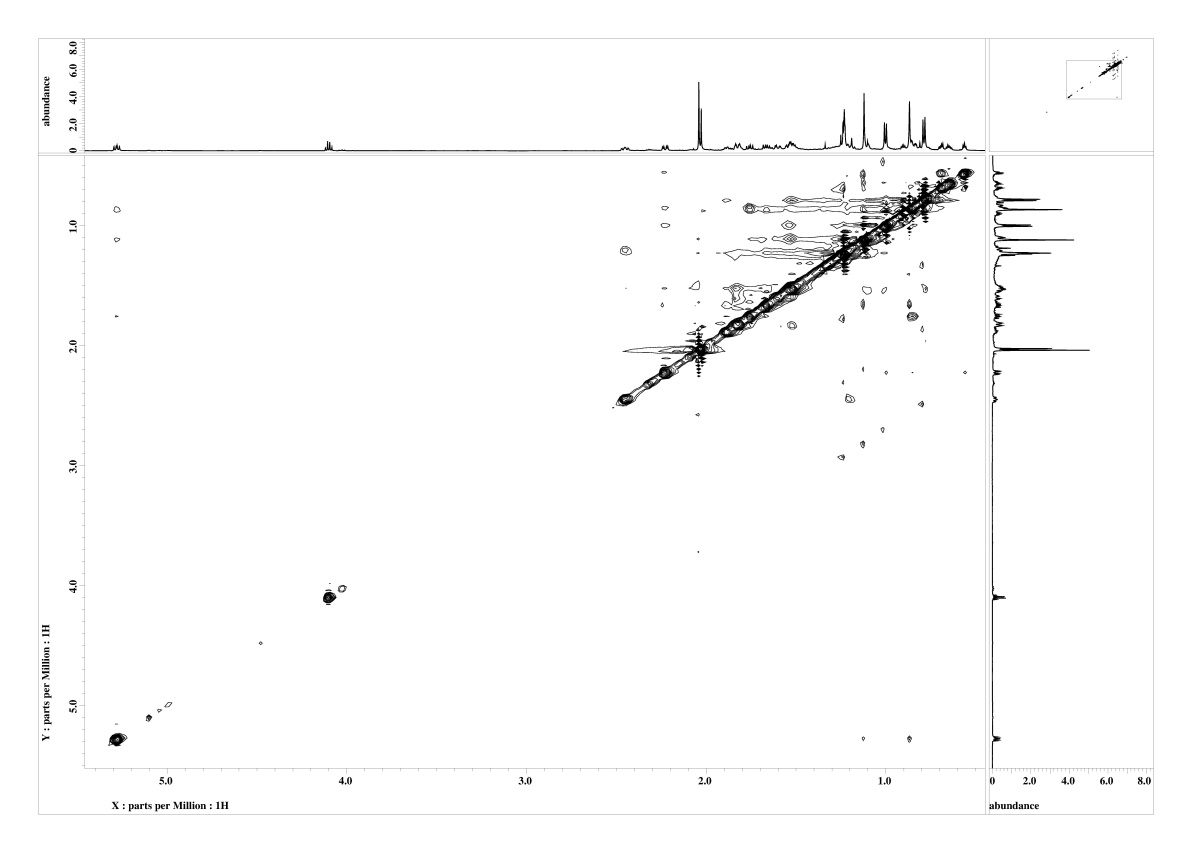


**Figure S7.** HRESIMS spectrum of compound **1**.


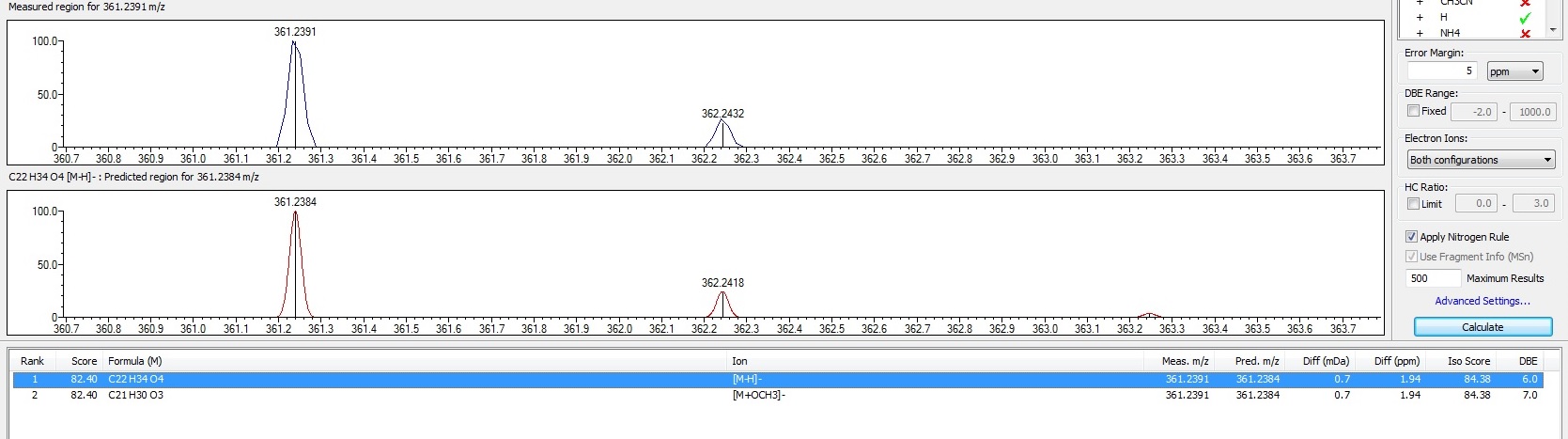

Supplement: Supplementary file 1 — Supplementary file1 (DOCX 594 kb) [file 13659_2020_232_MOESM1_ESM.docx]
